# HIV Tat Induces Expression of ICAM-1 in HUVECs: Implications for miR-221/-222 in HIV-Associated Cardiomyopathy

**DOI:** 10.1371/journal.pone.0060170

**Published:** 2013-03-28

**Authors:** Ming Duan, Honghong Yao, Guoku Hu, XianMing Chen, Amie K. Lund, Shilpa Buch

**Affiliations:** 1 Department of Pharmacology and Experimental Neuroscience, University of Nebraska Medical Center, Omaha, Nebraska, United States of America; 2 Key Laboratory for Zoonosis Research, Ministry of Education, Jilin University, Changchun, China; 3 Department of Medical Microbiology and Immunology, Creighton University School of Medicine, Omaha, Nebraska, United States of America; 4 Cardiopulmonary and Environmental Toxicology Department, Lovelace Respiratory Research Institute, Albuquerque, New Mexico, United States of America; National Institutes of Health, United States of America

## Abstract

Cardiac involvement is a well-documented complication of human immunodeficiency virus-1 (HIV-1) infection. Previous studies have demonstrated increased adhesion of monocytes to human vascular endothelial cells in HIV-infected individuals. HIV Tat protein, which is the transactivator of transcription (Tat), plays a key role in activating endothelial cells. In the present study, we demonstrated that exposure of HUVECs to HIV Tat protein resulted in induced expression of cell adhesion molecules specifically ICAM-1, leading to increased adhesion of monocytes to the endothelium. This effect of Tat was mediated through activation of mitogen-activated protein kinases and downstream transcription factor NF-κB. Increased expression of ICAM-1 was regulated by microRNA (miRNA) miR-221 and to some extent by miR-222, both of which are known to target ICAM-1. Functional inhibition of the respective miRNAs with anti-miR oligonucleotides resulted in induction of ICAM-1 protein in HUVECs. Furthermore, Tat-stimulated regulation of ICAM-1 via miR-221/-222 involved the NF-kB-dependent pathway. Functional implication and specificity of up-regulated ICAM-1 was confirmed using the ICAM-1 neutralizing antibody in the *in vitro* cell adhesion assays. These findings were further confirmed *in vivo* using the HIV transgenic (Tg) rats. These animals not only demonstrated increased expression of ICAM-1 mRNA, with a concomitant reduction in the expression of miR-221 in the aorta and heart, but also had increased expression of the ICAM-1 protein that was predominantly in the endothelial cell layer. Taken together, these findings implicate that Tat-mediated induction of ICAM-1 expression plays a critical role in monocyte adhesion observed in HIV-1-associated cardiomyopathies.

## Introduction

Several postmortem studies on AIDS patients have shown clear signs of cardiomyopathies [1]. Clinical studies on HIV-infected patients also provide evidence of progressive cardiac complications following HIV-1 infection [2], [3]. While the advent of anti-retroviral therapy has decreased the incidence of HIV-1 cardiomyopathy (HIVCM), its prevalence is actually on a rise. The mechanism(s) by which HIV-1 induces inflammation of the heart are not well understood, but are likely multifactorial in nature. HIV-1 viral protein Tat that is released by infected monocytes and taken up by neighboring cells has been shown to facilitate interaction of the monocyte with the endothelium [4], [5], resulting in the recruitment of monocytes into the extravascular tissue. This process, in turn, contributes to destruction of the tissue parenchyma and cellular architecture, a classic feature observed in patients with AIDS [5].

Accumulation of monocytes within the tissue leads to tissue damage and dysfunction. Monocyte adhesion is a dynamic, multistep process involving initial “rolling” of cells along the vessel endothelium in response to inflammatory mediators, arrest to endothelium and subsequent strong adhesion to the systemic vasculature [6]. Interaction of endothelial adhesion molecules with their cognate ligands on monocytes is critical for this process.

Up-regulation of adhesion molecules such as ICAM-1 and VCAM-1 is pivotal in the development of inflammatory responses. A previous study has demonstrated that interactions between ICAM-1 expressed on endothelial cells and circulating monocytes may be critical for the adhesion of these cells on the vascular endothelium [7]. HIV Tat is known to exhibit diverse functional aberrations on the endothelial cells [5]. For example, Tat-mediated impaired expression of adhesion molecules has been implicated as an early step in the development of cardiovascular disease associated with HIV-1-infection [5], [8], [9]. Although it has been documented that Tat mediated increase expression of ICAM-1 and VCAM-1 in HUVECs [4], detailed mechanisms underlying this process are not well elucidated.

MicroRNAs (miRNAs) are small RNA regulators (18–23 nucleotides) that play essential roles in a wide spectrum of biological processes [10], [11]. These molecules target mRNAs on the basis of complementary sequences between the miRNAs and the 3′-untranslated regions (3′UTRs) of the target mRNAs, resulting in suppression of cellular target genes by inducing either mRNA degradation and/or translational suppression [11]. Because miRNAs appear to provide quantitative regulation of genes, rather than “on-off” decisions, they can be envisioned as fine tuners of the cellular responses to external influences [12]. miRNAs regulate many disparate processes including regulation of expression of cell adhesion molecules. Previous reports indicate the role of miR-221 in suppressing ICAM-1 translation and regulating IFN-γ-induced ICAM-1 expression in human cholangiocytes [13]. However, the role of these miRNAs in the context of HIV-1 infection in HUVECs has not been yet determined.

The present study was aimed at exploring the molecular mechanisms by which Tat mediates induction of ICAM-1 in vascular endothelial cells. Understanding the regulation of ICAM-1 expression by Tat may provide insights into the development of therapeutic targets aimed at blocking inflammation in the heart of HIV-1 individuals.

## Materials and Methods

### Reagents

IκB kinase-2 (IKK2)/NF-kB inhibitor SC514 was purchased from Sigma Chemicals (St. Louis, MO, USA). Specific inhibitors of MEK1/2 (U0126), JNK (SP600125) and p38 (SB 203580) were purchased from Calbiochem (San Diego, CA, USA). The concentrations of these inhibitors were based on the concentration-curve study in our previous reports [14]. Treatment of human umbilical vein endothelial cells (HUVECs) with pharmacological inhibitors (U0126∶20 µM; SP600125∶20 µM; SB203580∶20 µM; SC514∶10 µM) involved pre-treatment of cells with the respective inhibitors for 1 h followed by exposure to Tat.

### Animals

For the endpoints from animal studies described herein we used 9- month old HIV transgenic male rats [HIV Tg rat, Hsd: HIV-1 (F344) Harlan] (n = 6) and 9-month old F344 male rats (as age and background-matched controls) (n = 6). HIV Tg rats express viral genes in the lymph nodes, spleen, thymus, and blood, suggesting rat cyclin T is functional with Tat; however, the animals are noninfectious due to the functional deletion of *Gag* and *Pol* within the HIV-1 provirus (thus carries only 7 of the 9 HIV genes) [15]. Rats were housed in pairs in microisolator cages within an Association for Assessment and Accreditation of Laboratory Animal Care International-approved rodent housing facility for the entirety of the study, which maintained constant temperature (20–24°C) and humidity (30–60% relative humidity). Rats had access to chow and water *ad libitum* throughout the study period. All animal procedures were approved by the Lovelace Respiratory Research Institute’s Animal Care and Use Committee and conform to the Guide for the Care and Use of Laboratory Animals published by the US National Institutes of Health (NIH Publication No. 85-23, revised 1996). All animals were euthanized by exsanguination under anesthesia (5% isoflurane by mask). Tissues were dissected and immediately snap frozen [a portion of each the heart and aorta were embedded in Tissue Tek® O.C.T. (VWR Scientific, West Chester, PA, USA) medium for histology and frozen on dry ice], and stored at -80°C until used for analysis.

### Cell Culture

HUVECs, originally acquired from Lonza (Walkersville, MD), were obtained from Dr. Mukesh K. Jain’s lab (Case Western Reserve University, Cleveland, Ohio, USA) and cultured in EBM-2 media as described in previous report [16].

### Flow Cytometry

HUVECs treated with Tat were collected in cold PBS and EDTA (5 mM) followed by incubation with anti-ALCAM (3A6, 1∶100; BD Biosciences, San Jose, CA, USA), anti-ICAM-1 (HA58, 1∶1000; BD Biosciences, San Jose, CA, USA) and anti-VCAM-1(51-10C9, 1∶100; BD Biosciences, San Jose, CA, SA) antibodies. LSR II (BD Biosciences, San Diego, CA, USA) was used for fluorescence acquisition and data were analyzed with FACSDiva software (BD Biosciences, San Diego, CA, USA). The samples were gated using a forward scatter and side scatter gate eliminating debris. The fluorescent parameters were set based on the unstained controls.

### Reverse Transcription and Real-time PCR

Total RNA was extracted with Trizol reagent (Invitrogen, Carlsbad, CA, USA) according to the manufacturer’s instructions, as previously reported [14]. For *in vivo* RNA isolation endpoints, total RNA was isolated from the right superior lobe using an AllPrep DNA/RNA/protein kit (Qiagen, Valencia, CA, USA). cDNA was synthesized from total RNA in a 20 µl final reaction volume using a Verso cDNA Synthesis Kit (Thermo Fisher Scientific, Waltham, MA, USA). The mixture was heated at 42°C for 1 h and then cooled to 4°C. Real-time PCR was performed with gene-specific primers in the ABI 7900 (Applied Biosystems, Foster City, CA, USA). The following primers were used for the PCR reactions: Rat-ICAM-1 FP: TTCAAGCTGAGCGACATTGG; RP: CGCTCTGGGAACGAATACACA; Human-ICAM-1 FP: CACAGTCACCTATGGCAACGA; RP: TGGCTTCGTCAGAATCACGTT;GADPH FP:GCCAAAAGGGTCATCATCTC; RP: GGCATGGACTGTGGTCATGAG.

Samples were run in triplicate and results for each run were averaged. ΔCT (change in threshold cycle) was calculated by subtracting the CT of the GAPDH control gene from the CT value of the gene of interest and mean normalized gene expression was calculated as previously described [17]. Results are expressed as normalized gene expression as percentage of GAPDH control.

For analysis of miR-221/-222, total RNA was isolated from cells with Trizol reagent (Invitrogen, Carlsbad, CA, USA) according to the manufacturer’s instructions. Comparative real-time PCR was performed by using the TaqMan Universal PCR Master Mix (Applied Biosystems, Foster City, CA, USA). Specific primers and probes for mature miR-221, -222 and snRNA RNU6B were obtained from Applied Biosystems. All reactions were run in triplicate. The amount of miR-221/-222 was obtained by normalizing to snRNA RNU6B and relative to control as previously reported [18], [19].

### Overexpression and knock Down of miRNAs

To manipulate the cellular functions of miR-221/-222 in HUVEC cells, we utilized an antisense approach to inhibit miR-221/-222 function and transfection of cells with miR-221/-222 precursor to increase miR-221/-222 expression. Briefly, HUVECs were grown to 70% confluency and transfected with either anti-miR-221/-222 (antisense 2-methoxy oligonucleotide to miR-221/-222, Ambion, Austin, TX, USA) or the miR-221/-222 precursor (Ambion, Austin, TX, USA) using the lipofectamine 2000 reagent (Invitrogen, Carlsbad, CA, USA) followed by analysis of ICAM-1 expression and cell adhesion.

### Western Blot Analysis

Treated cells or tissue were lysed using the Mammalian Cell Lysis kit (Sigma, St. Louis, MO, USA) and the NE-PER Nuclear and Cytoplasmic Extraction kit (Pierce, Rockford, IL, USA) and quantified using the micro BCA Protein Assay kit (Pierce, Rockford, IL, USA). Equal amounts of the corresponding proteins were electrophoresed in a sodium dodecyl sulfate-polyacrylamide gel (10–12%) under reducing conditions followed by transfer to PVDF membranes. The blots were blocked with 5% non-fat dry milk in phosphate buffered saline. Western blots were then probed with antibodies recognizing the ICAM-1, ERK1/2, JNK, p38,NF-κB p65 (1∶200; Cell Signaling Technology, Danvers, MA, USA) and β-actin (1∶4000; Sigma, St. Louis, MO, USA). The secondary antibodies were alkaline phosphatase conjugated to goat anti mouse/rabbit IgG (1∶5000). Signals were detected by chemiluminescence and imaged on the FLA-5100 (Fujifilm, Valhalla, NY, USA) digital image scanner; densitometry was performed utilizing Image J software (NIH) [20].

### Cell Adhesion Assay

Monocytes were obtained from unidentified HIV-1/HIV-2/Hepatitis B seronegative donor from Blood, Transfusion & Tissue Service at UNMC, and separated by countercurrent centrifugal elutriation as previously described [21]. Freshly elutriated monocytes were cultured in DMEM containing 10% heat-inactivated human serum, 2 mmol/l L-glutamine (Invitrogen, Carlsbad, CA, USA), 100 mg/ml gentamicin, and 10 mg/ml ciprofloxacin (Sigma, St. Louis, MO, USA).

For monocyte adhesion, HUVECs were seeded on 96-well plates at a density of 2.5×10^4^cells/well and following confluency were treated with different concentrations of Tat for 24 h as described previously [22]. In parallel, monocytes (5×10^6^ cells/ml) were also labeled with 10 µM cell tracker green (Invitrogen, Carlsbad, CA, USA) for 10 min, following which, monocytes were then incubated with HUVECs for 15 min at 37°C and rinsed thrice with PBS to remove the nonadherent monocytes. The fluorescence intensity of adherent monocytes was measured using a Synergy Mx fluorescence plate reader (Bio-Tek Instruments, Winooski, VT, USA).

### Immunofluorescence

Sections of aorta or heart (6 µm) were incubated with 10% normal goat serum for 30 min at RT, washed in PBS, and then incubated with 250 µl per section of mouse monoclonal ICAM-1 (1∶1000; Abcam, Cambridge, MA, USA) primary antibody diluted in buffer [1 part 5% blocking solution (0.5 ml Normal Rabbit Serum in 10 ml 3% w/v Bovine Serum Albumin) and 4 parts PBS] for 1 h at RT, and then rinsed 3 times with PBS. Tissue section slides were then incubated in 250 µl/per slide of secondary antibody Alexa Flour 594 (goat anti-mouse) (1∶2000; Vector Laboratories, Biovalley SA, Marne la Vallée, France) with DAPI (0.5 µg/ml) in the dark for 1 h at RT. Slides were subsequently rinsed thrice in PBS, and coverslipped with Aqueous Gel Mount (Sigma, St. Louis, MO, USA). Slides were imaged by fluorescent microscopy at 10x, 40x, and 63x using the appropriate excitation/emission filters, digitally recorded, and analyzed by image histogram, quantifying total red fluorescence, using Image J software (NIH). A minimum of 2 locations on each section (2 sections per slide), 3 slides and n = 3–5 per group were processed/analyzed.

### Statistical Analysis

Data were expressed as mean ± standard deviation (SD) from at least three separate experiments. Significance of differences between groups was determined by Student’s *t* test. Values of P<0.05 were taken as statistically significant.

For *in vivo* study data expressed as mean ± SD. A *t*-test was used to compare between HIV Tg rat and F344 rat groups for real time PCR, Western blot, and immunofluorescence endpoint analysis, using Sigma Plot (v.10.0, Systat Inc., CA, USA). A p<0.05 was considered statistically significant.

## Results

### Tat-mediated up-regulation of ICAM-1 in HUVECs

**Figure 1:**
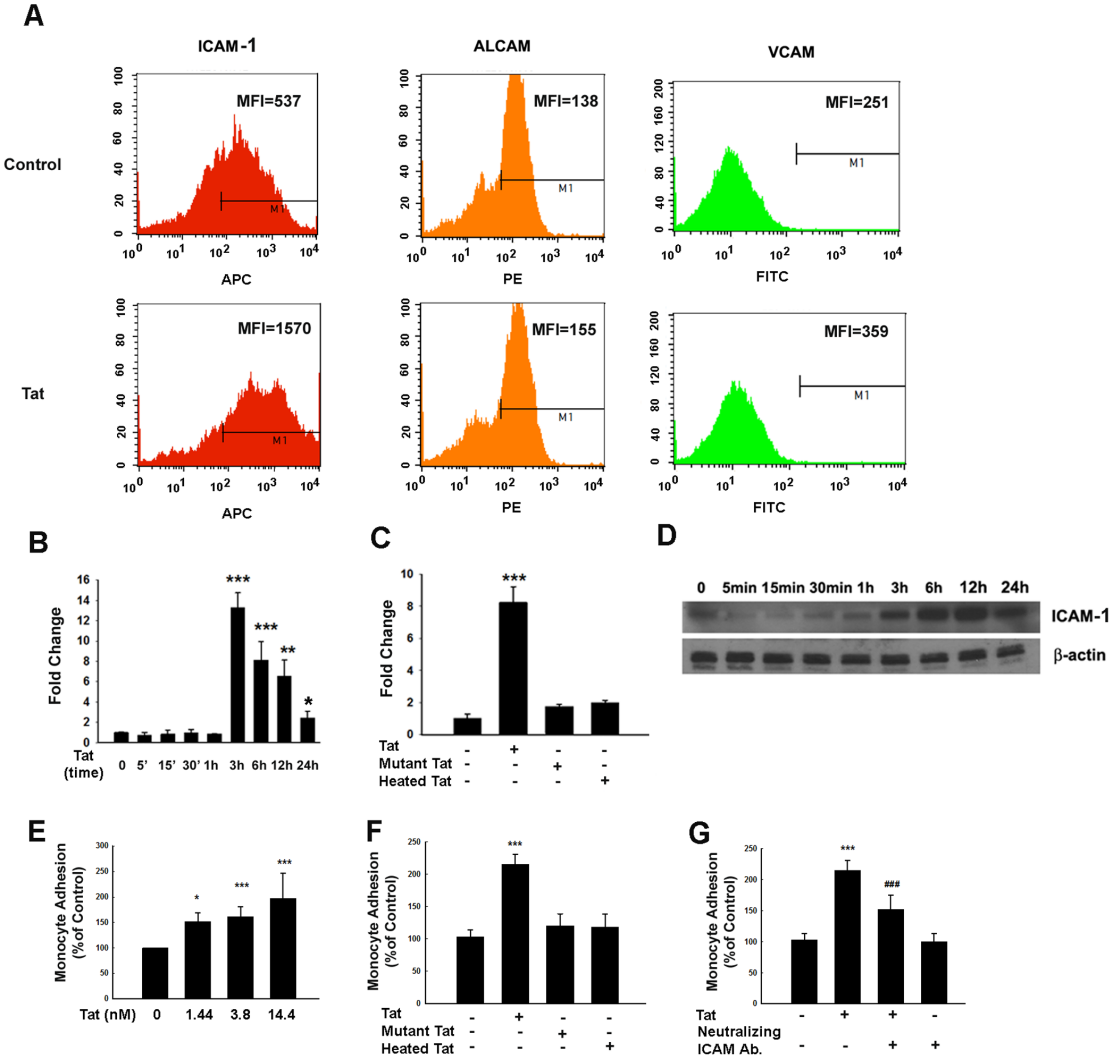
Tat-mediated induction of ICAM-1 expression in HUVECs. (**A**) Flow cytometric analysis indicating Tat (14.4 nM)-mediated changes in ALCAM, ICAM & VCAM in HUVECs. (**B**) Exposure of HUVECs to Tat induced time-dependent induction of ICAM-1 mRNA expression by real-time RT-PCR. (**C**) Effect of Tat, mutant- or heated-Tat on the ICAM-1 mRNA expression by real-time RT-PCR. (**D**) Western blot analysis demonstrated time-dependent induction of ICAM-1 by Tat (14.4 nM) in HUVECs. (**E**) Exposure of HUVECs to varying concentrations of Tat induced monocyte adhesion. (**F**) Effect of Tat, mutant- or heated-Tat on the monocyte adhesion. (**G**) Neutralizing antibody of ICAM-1 abrogated monocyte adhesion induced by Tat. HUVECs were exposed to Tat for 12 h (14.4 nM) followed by cell adhesion assay. All the data are presented as mean ± SD of three independent experiments. *p<0.05; **p<0.01; ***p<0.001 vs control; ^###^p<0.001 vs Tat-treated group.

Since HIV-1 infection is known to alter the interaction of monocytes with human vascular endothelial cells [23] by regulating expression of adhesion molecules [6], in the present study we sought to assess the effect of Tat on the expression of cell adhesion molecules. Using flow cytometry, it was demonstrated that under normal condition HUVECs expressed almost equal levels of both ICAM-1 and activated leukocyte cell adhesion molecule (ALCAM), and lower levels of VCAM-1 (Fig. 1A). Intriguingly, activation of cells with Tat elicited a robust up-regulation of ICAM-1, and a weaker induction of both ALCAM and VCAM-1 (Fig. 1A). We next sought to examine the time course of ICAM-1 induction. Tat-mediated induction of ICAM-1 mRNA in HUVECs was observed as early as 3 h following treatment and was significantly up-regulated even at 24 h post-treatment (Fig. 1B). However, heat-inactivated or mutant Tat failed to exert any significant effect on ICAM-1 expression (Fig. 1C). This finding was further confirmed by Western blot analysis demonstrating increased time-dependent expression of ICAM-1 following Tat exposure (Fig. 1D).

Since cell adhesion molecules are critical for monoctye adhesion, the next step was to examine the effect of Tat on monocyte adhesion. HUVECs were exposed to varying concentrations of Tat protein for 24 h followed by assessment of human monocyte adhesion using the *in vitro* assay. As shown in Fig. 1E, there was a concentration-dependent effect of Tat on monocyte adhesion with increasing concentrations of Tat (1.44, 3.6 and 14.4 nM, or 20, 50, 200 ng/ml), resulting in increased monocyte adhesion (29.5% [p<0.05]; 46.4% [p<0.001] and 74.5% [p<0.001]) respectively, with a maximal response at 14.4 nM (Fig. 1E). The concentration of Tat in the cerebral spinal fluid (CSF) has been reported to be 16 ng/ml [24]. However, the concentration of Tat in the brain remains unknown but is expected to be much higher than in the CSF [25]. The Tat concentration utilized in this *in vitro* study is in the physiological range [26], [27]. Treatment of HUVECs with either heat-inactivated or mutant Tat did not exert any significant effect on monocyte adhesion, as expected (Fig. 1F).

Have determined the dose and time-course of Tat-mediated induction of ICAM-1, the next step was to explore the specificity of ICAM-1 in this process. HUVECs were pretreated with a neutralizing ICAM-1 antibody, followed by assessment of monocyte adhesion in the presence of Tat. In the presence of the ICAM-1 antibody, Tat failed to induce significant monocyte adhesion (Fig. 1G), thereby underpinning the role of ICAM-1 in monocyte adhesion.

### Tat-induced Expression of ICAM-1 Involves MAPK (ERK1/2, JNK, p38) and Downstream NF-kB Pathways

**Figure 2:**
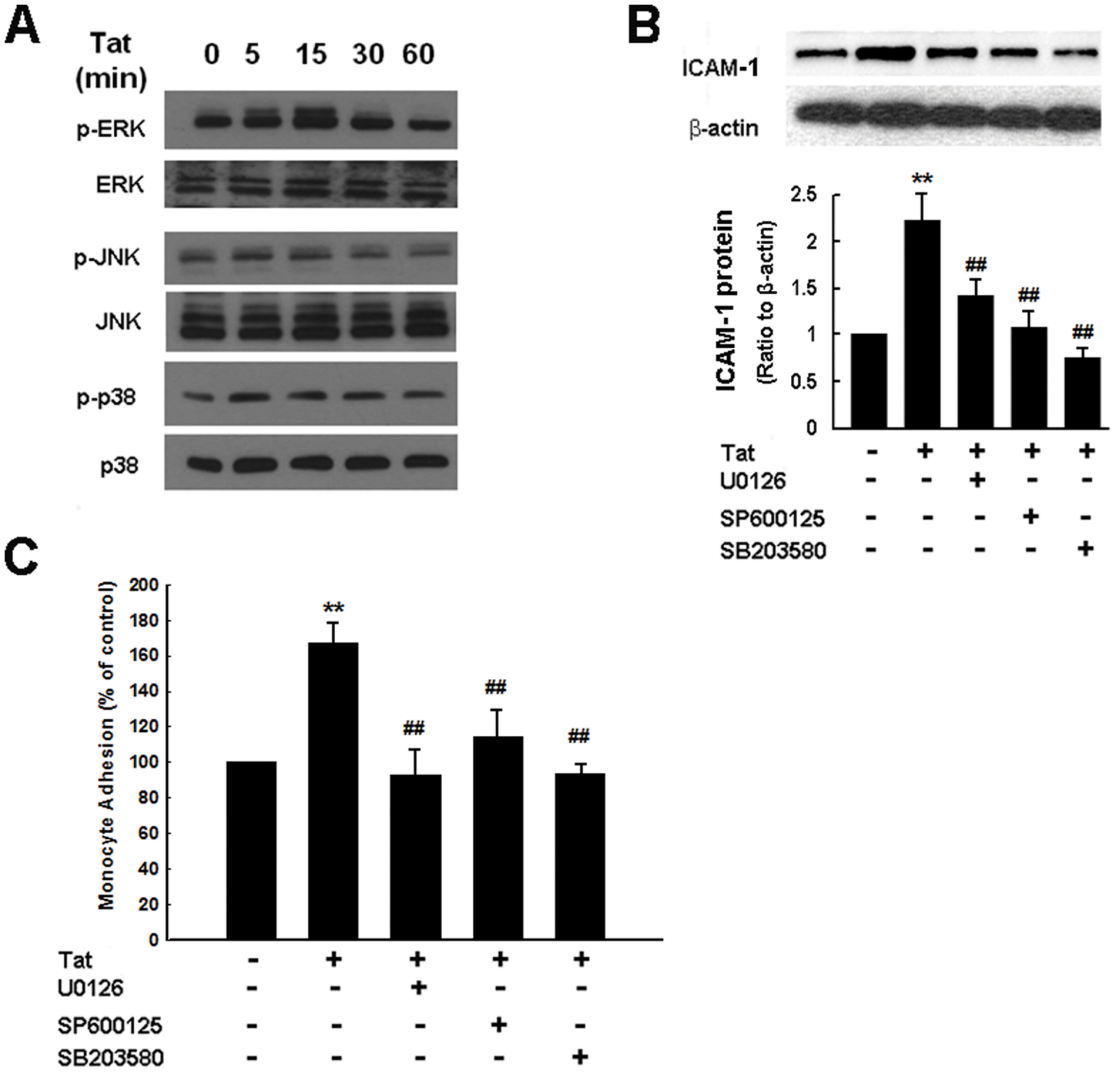
Tat-mediated induction of ICAM-1 expression involves MAPK signaling pathways. (**A**) Western blot analysis demonstrated time-dependent activation of ERK, JNK and p38 by Tat in HUVECs. (**B**) Inhibition of the ERK, JNK and p38 MAPK pathways by MEK1/2 (U0126, 20 µM), JNK (SP600125, 20 µM) and p38 (SB203580, 20 µM) inhibitors resulted in amelioration of Tat-mediated induction of ICAM-1 expression. (**C**) Pharmacological inhibition of MAPK pathways by MEK1/2 (U0126, 20 µM), JNK (SP600125, 20 µM) and p38 (SB203580, 20 µM) resulted in amelioration of Tat-mediated induction of monocyte adhesion. All the data are presented as mean ± SD of three independent experiments. **p<0.01 vs control; ^##^p<0.01 vs Tat-treated group.

MAPK kinase pathways play critical roles in expression of adhesion molecules [28]. We next sought to examine the involvement of these pathways in Tat-mediated induction of ICAM-1. Treatment of HUVECs with Tat resulted in a time-dependent increase in phosphorylation of MAPK (Fig. 2A) and reciprocally, pretreatment of cells with inhibitors specific for MEK1/2 (U0126), JNK (SP600125) or p38 (SB203580) resulted in amelioration of Tat-mediated induction of ICAM-1, thereby underscoring the roles of these kinases in this process (Fig. 2B). The next step was to link Tat-mediated activation of MAPKs signaling pathways with monocyte adhesion. As shown in Fig. 2C, inhibitors specific for MAPKs blocked Tat-induced monocyte adhesion, further supporting the notion that in HUVECs sequential activation of MAPKs pathways results in up-regulation of ICAM-1 and subsequent monocyte adhesion.

**Figure 3:**
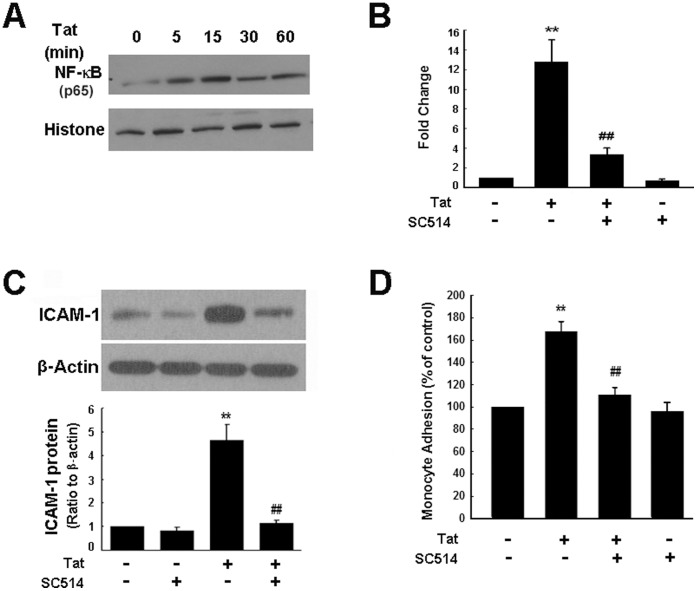
Tat-mediated induction of ICAM-1 expression is NF-κB-dependent manner. (**A**) Tat mediated time-dependent activation of NF-κB in HUVECs. Pre-treatment of HUVECs with the IKK2/NF-κB inhibitor SC514 (10 µM) abrogated Tat-induced ICAM-1 expression as shown by real-time RT-PCR (**B**) and Western blot (**C**) analyses. (**D**) Monocyte adhesion induced by Tat was inhibited in HUVECs by pre-treatment of cells with IKK2 inhibitor SC514 (10 µM). HUVECs were exposed to Tat for 12 h followed by cell adhesion assay. All the data are presented as mean ± SD of three independent experiments. **p<0.01 vs control; ^##^p<0.01 vs Tat-treated group.

Members of the NF-κB family are considered to play essential roles in ICAM-1 expression [29], [30]. We therefore next examined the role of NF-kB in the induction of ICAM-1. Treatment of HUVECs with Tat resulted in translocation of NF-κB p65 into the nucleus (Fig. 3A). Pretreatment of cells with IKK2 inhibitor SC514 abrogated Tat-induced ICAM-1 expression at both the mRNA (Fig. 3B) and protein (Fig. 3C) levels, thereby confirming a role for NF-κB p65 in Tat-mediated induction of ICAM-1. Moreover, we determined the role of activated NF-kB pathway in Tat-mediated adhesion of monocytes as evidenced by the fact that pretreatment of cells with SC514 blocked Tat-induced monocyte adhesion (Fig. 3D), further lending support to the notion that in HUVECs sequential activation of NF-kB pathway leads to up-regulation of ICAM-1 and subsequent monocyte adhesion.

### Tat-induced Expression of ICAM-1 in HUVECs Involves miR-221/-222 Suppression

**Figure 4:**
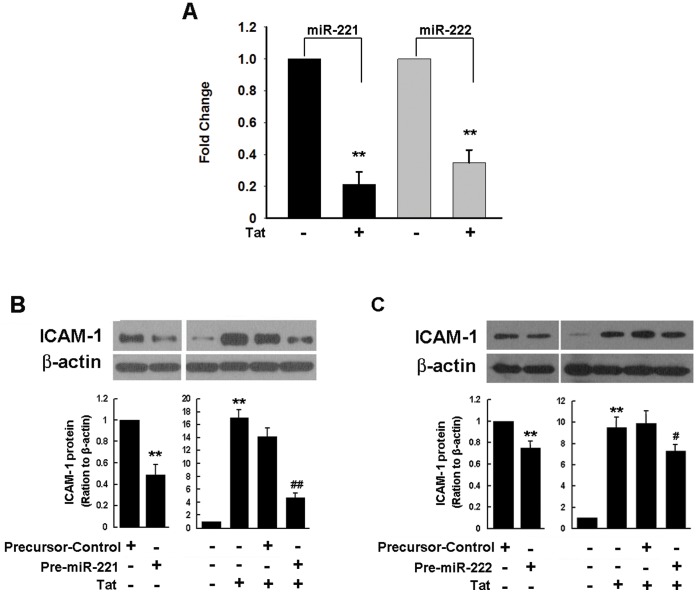
Tat-induced expression of ICAM-1 in HUVECs involves miR-221/-222 suppression. (**A**) Effect of Tat on the miR-221/-222 by real-time RT-PCR in HUVECs after 6 h exposure to Tat. RNU6B (U6) was used as the control. (**B**) Functional overexpression of miR-221/-222 (50 nM) decreased ICAM-1 protein expression. HUVECs transfected with miR-221 or miR-222 precursor or a precursor control for 24 h were exposed to Tat (14.4 nM) for 12 h followed by western blot analysis for ICAM-1. All the data are presented as mean ± SD of three independent experiments. **p<0.01 vs control; ^#^p<0.05, ^##^p<0.01 vs Tat-treated group.

Recent studies suggest that ICAM-1 is a target of miR-221/-222, both of which regulate ICAM-1 expression in response to inflammatory stimuli [13], [31]. To examine whether posttranscriptional regulation by miR-221/-222 was critical for Tat-induced expression of ICAM-1, HUVECs were treated with Tat and assessed for expression of miR-221/-222 by real-time PCR. Following Tat-mediated stimulation of HUVECs, there was a significant decrease in the expression levels of both miR-221/-222 in HUVECs following Tat stimulation for 12 h, as assessed by real-time PCR (Fig. 4A).

Next, in order to test the role of miR-221/-222 in Tat-mediated induction of ICAM-1, HUVECs were transfected with miR-221/-222 precursors for 24 h, followed by exposure to Tat for 12 h, with subsequent assessment of ICAM-1 protein expression by Western blot. As shown in Fig. 4B and 4C, precursors of both miR-221/-222 significantly blocked Tat-induced ICAM-1 protein expression in HUVECs. Transfection of cells with a precursor control sequence, however, did not inhibit Tat-mediated induction of ICAM-1.

### Tat-mediated Decrease of miR-221/-222 Expression Involves NF-kB Pathway

**Figure 5:**
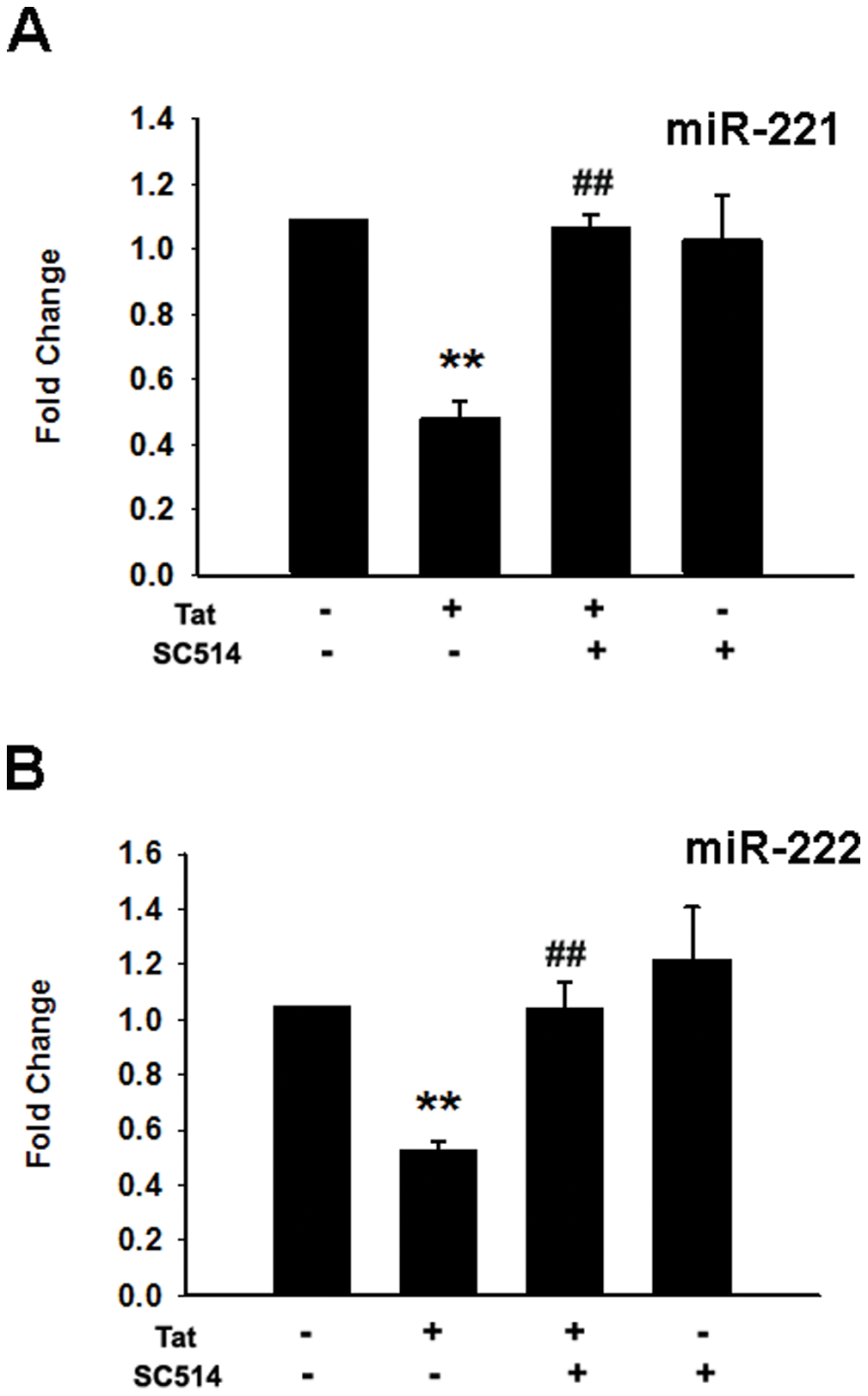
Tat-mediated suppression of miR-221/-222 is NF-κB-dependent. Pre-treatment of HUVECs with the IKK2/NF-κB inhibitor SC514 (10 µM) abrogated Tat-mediated down-regulation of miR-221/-222 expression. Total RNA was isolated, and the expression of miR-221/-222 was quantified by real-time RT-PCR. RNU6B (U6) was used as the control. All the data are presented as mean ± SD of three independent experiments. **p<0.01 vs control; ^##^p<0.01 vs Tat-treated group.

Having determined the role of NF-kB in Tat-induced expression of ICAM-1, we next sought to examine its role in Tat-mediated down-regulation of miR-221/-222. Cells were pretreated with SC514 for 1 h followed by additional incubation for 12 h in the presence or absence of Tat and assessed for expression of miR-221/-222 by real-time PCR analysis. A significant decrease in miR-221/-222 levels was detected in Tat-treated HUVECs, which was significantly ameliorated in cells pretreated with SC514 (Fig. 5A, B).

### MiR-221/-222 Regulated Expression of ICAM-1 Plays a Role in Monocyte Adhesion

**Figure 6:**
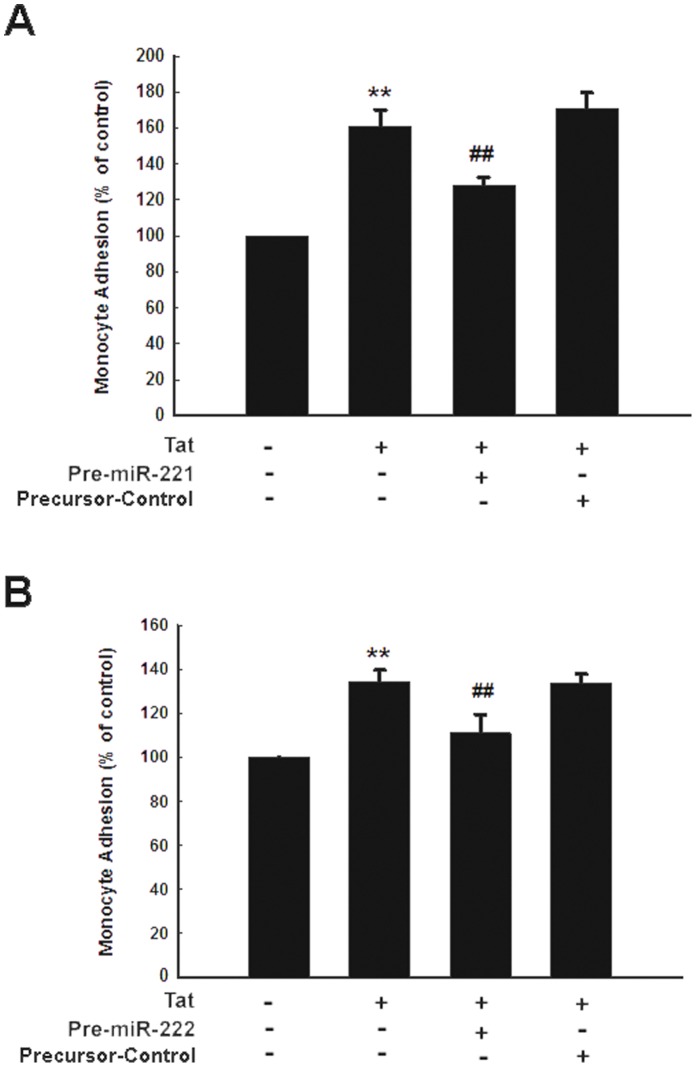
Overexpression of miR-221 or miR-222 inhibits monocyte adhesion induced by Tat in HUVECs. Transfection of HUVECs with miR-221 or miR-222 precursor abrogated Tat-induced monocyte adhesion. HUVECs were transfected with miR-221 or miR-222 (50 nM) precursors for 24 h, then exposed to Tat (14.4 nM) for 12 h followed by monocyte adhesion assay. All the data are presented as mean ± SD of three independent experiments. **p<0.01 vs control; ^##^p<0.01 vs tat-treated group.

Over-expression of adhesion molecule such as ICAM-1 on endothelial cells is known to directly contribute to increased monocyte adhesion on the endothelium. In order to determine the functional relevance of miR-221/-222-regulated expression of ICAM-1 in HUVECs, monocyte adhesion assays were performed in the presence of HUVECs transfected with miR-221/-222 precursors. As shown in Fig. 6A and B, there was significant reduction of monocyte adhesion in cells transfected with either of the precursors. These data further corroborate that Tat-mediated suppression of miR-221/-222 is involved in expression of ICAM-1 in HUVECs and also influences monocyte adhesion.

### Up-regulation of ICAM-1 Expression in the Aorta from HIV Transgenic (Tg) Rats

**Figure 7:**
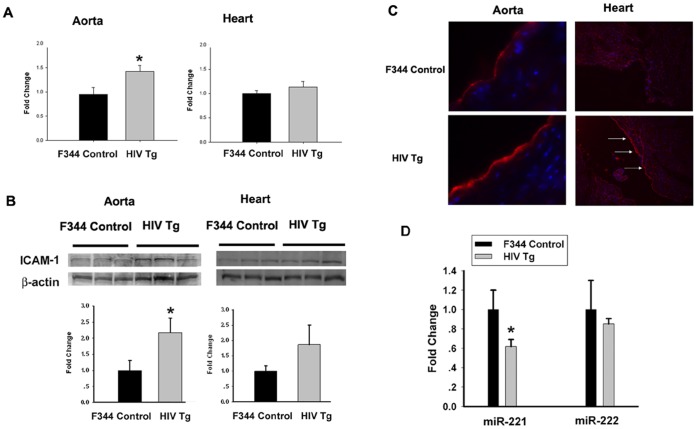
Expression of ICAM-1 in HIV Tg and F344 control rats. (**A**) Expression in HIV Tg and F344 control rat in the aorta and heart by real time RT-PCR; (**B**) ICAM-1 protein expression in the aorta and heart by western blot analysis. Representative immunoblot of ICAM-1 was presented in the upper panel. Densitometric analysis of ICAM-1/β-actin. N = 3. *p<0.05 vs F344 control; (**C**) ICAM-1 immunofluorescence in the aorta and heart. ICAM-1 expression is indicated by red fluorescence, blue fluorescence is nuclei staining (DAPI), and arrows indicate increased ICAM-1 fluorescence. (**D**) miR-221/-222 mRNA expressions in HIV Tg vs. F344 control (WT) aortas. *p<0.05 compared to F344 controls.

To validate the cell culture findings *in vivo*, next step was to examine expression of ICAM-1 RNA and protein both in isolated aorta as well as the heart tissues isolated from HIV Tg rats that express 7 of the 9 HIV proteins including Tat. As shown Fig. 7A, there was a significant increase in expression of ICAM-1 mRNA in the aorta of HIV Tg rats compared with the wide type (WT) animals. In the heart of Tg animals, while there was a trend towards increased ICAM-1 mRNA expression compared with the WT rats, the difference was not statistically significant. Immunofluorescence findings herein thus suggest that in the hearts of HIV transgenic rats, ICAM-1 is up-regulated in the endocardium (endothelial cells), leading to functional consequences of increased monocyte adherence in the aorta/heart. Intriguingly, homogenates of the aorta and heart also demonstrated increased expression of ICAM-1 when assessed by real-time RT-PCR and Western blot as shown in Figs. 7B and C. Furthermore, consistent with the *in vitro* findings, expression of miR-221, but not miR-222, was decreased in aorta isolated from HIV Tg rats, compared with the WT rats (Fig. 7D).

## Discussion

Numerous studies have shown that HIV infection is an independent risk factor for cardiovascular disease [2], [32], [33]; however, the mechanism(s) by which HIV-1 induces cardiovascular disease (CVD) are not well understood and are likely multifactorial. HIVCM are accompanied by traditional cardiovascular risk factors, including endothelial dysfunction, chronic inflammation, lipid disorders, as well as proposed direct HIV-1 infection of the endothelium [34], [35], [36]. Additionally, several HIV viral proteins have been implicated in causing HIV-associated cardiovascular disease through direct interactions at the endocardium, resulting in apoptosis, alterations in endothelial permeability and endothelial cell activation, ultimately culminating into induced expression of adhesion molecules [36], [37], [38]. However, the detailed mechanisms underlying these processes remain unclear.

Adhesion molecules such as ICAM-1 are known to mediate leukocyte (and platelet)-endothelial cell interactions in the vasculature under both physiological and pathological conditions. Importantly, ICAM-1 has been shown to play an essential role in the development of vascular inflammation and endothelial dysfunction [39] that is associated with the pathogenesis of multiple cardiovascular diseases including atherosclerosis, coronary artery disease, myocarditis, and hypertrophic cardiomyopathy [40], [41]. In the present study, it was demonstrated that exposure of HUVECs to Tat leads to the induction of both ICAM-1 mRNA and protein. To delve deeper into the molecular mechanisms of Tat-mediated induction of ICAM-1 expression, the intercellular signaling events such as MAPK kinases were assessed. Using both the pharmacological and genetic approaches, we examined the activation of MAPK kinase pathways in Tat-mediated induction of ICAM-1. These findings are consistent with the previous reports implicating the role of these signaling pathways in the induction of ICAM-1 [30], [42]. MAPKs followed by the activation of NF-kB participate in the intracellular signal transduction and production of adhesion molecules in various cell systems [30], [43]. Consistent with these findings, the role of MAPKs and p65/RelA nuclear translocation were also demonstrated in Tat-mediated induction of ICAM-1expression.

Recent years have seen a splurge in publications on the gene regulation by miRNAs, small RNA regulators that play essential roles in disparate biological processes [10], [11]. Recent reports indicate the role of miR-221/-222 in suppressing ICAM-1 expression [13], [31]. In the present study, for the first time, we report the role of Tat in modulating the regulation of ICAM-1 via miR-221/-222 in HUVECs. We have demonstrated that the downregulation of miR-221/-222 involves activation of NF-kB. These findings complement previous studies on the role of miR-221/-222 in suppression for ICAM-1 translation, as evidenced by the fact that miR-221/-222 targeted ICAM-1 3′UTR and suppressed ICAM-1 translation [13]. Further confirmation of the role of miR-221/-222 was carried out by overexpressing miR-221/-222, which significantly decreased Tat-induced monocyte adhesion, underpinning the role of miR-221/-222 in regulating of ICAM-1 expression with concomitant monocyte adhesion.

Functional significance of Tat-induced ICAM-1 was examined in an *in vitro* model of cell adhesion. Treatment of HUVECs with Tat resulted in increased monocyte adhesion, and this effect was significantly inhibited by pre-treating cells with the neutralizing antibody specific for ICAM-1, thereby underpinning the role of ICAM-1 in monocyte adhesion induced by Tat. Further validation of these cell culture findings was carried out *in vivo* in a well-established HIV transgenic rat model that expresses 7 of the 9 HIV genes including Tat. Intriguingly, a recent report has demonstrated increased cardiomyopathy in HIV Tg animals compared to the WT controls as evidenced by elevated right ventricular systolic pressure and right ventricular hypertrophy [44]. Based on this as well as on our cell culture findings that Tat induced ICAM-1 expression in HUVECs, we sought to examine the expression of ICAM-1 protein both in the aorta as well as the heart tissue of these adult HIV Tg rats. The rationale behind this being that the accumulation of Tat in these adult animals could lead to increased ICAM-1 expression in the heart and aorta of these animals, thus leading to cardiac complications. As shown there was a significant elevation in ICAM-1 RNA and protein expression in the aorta, and to a lesser degree in the hearts of HIV-1 Tg rats, compared with the WT controls. Interestingly, immunofluorescence staining of the hearts revealed a notable increase in ICAM-1 expression specific to the endothelial layer (endocardium) of the heart of the HIV Tg rats. Furthermore, similar to our cell culture findings there was a significant decrease in the RNA expression of miR-221 in the aortas isolated from HIV Tg rats compared with the WT controls. While miR-222 RNA was also downregulated in the aortas of Tg rats, the reduced level of expression was not significant compared to controls. Taken together these findings underpin the role of miR-221 in regulating expression of ICAM-1. These findings are in concordance with a similar report identifying the role of miR-221 in suppression of ICAM-1 translation [13].

In summary, our findings have identified a detailed molecular pathway of Tat-mediated induction of ICAM-1, involving activation of MAPK pathways leading to translocation of NF-κB into the nucleus and ultimately resulting in increased ICAM-1. Furthermore, Tat-mediated expression of ICAM-1 played a vital role in monocyte adhesion, leading to increased recruitment of inflammatory cells onto the vessel endothelium. These findings not only have implications for HIV-1-infected individuals that have an increased risk of CVD, but also infected individuals that are treated with ART. In this latter population, increased longevity due to ART usage has resulted in increased prevalence of CVD, which is becoming an increasingly important health burden [45], [46], [47], [48]. In light of our findings and the published report that exposure of human aortic endothelial cells to combinations of protease inhibitors and nucleoside reverse transcriptase inhibitors resulted in increased expression of ICAM-1 VCAM-1, and endothelial-leukocyte adhesion molecule [49], it can be envisioned that HIV infection and ART together can lead to higher risk of development of CVD. Therapies provided to infected patients must thus be administered with caution to avoid drug-mediated effects on CVD.

## References

[1] Barbaro G, Lipshultz SE (2001) Pathogenesis of HIV-associated cardiomyopathy. Ann N Y Acad Sci 946: 57–81. 11762996 10.1111/j.1749-6632.2001.tb03903.x

[2] Barbaro G, Di Lorenzo G, Grisorio B, Barbarini G (1998) Incidence of dilated cardiomyopathy and detection of HIV in myocardial cells of HIV-positive patients. Gruppo Italiano per lo Studio Cardiologico dei Pazienti Affetti da AIDS. N Engl J Med 339: 1093–1099. 9770555 10.1056/NEJM199810153391601

[3] Starc TJ, Lipshultz SE, Kaplan S, Easley KA, Bricker JT, et al (1999) Cardiac complications in children with human immunodeficiency virus infection. Pediatric Pulmonary and Cardiac Complications of Vertically Transmitted HIV Infection (P2C2 HIV) Study Group, National Heart, Lung, and Blood Institute. Pediatrics 104: e14. 10429132 10.1542/peds.104.2.e14 PMC4358844

[4] Dhawan S, Puri RK, Kumar A, Duplan H, Masson JM, et al (1997) Human immunodeficiency virus-1-tat protein induces the cell surface expression of endothelial leukocyte adhesion molecule-1, vascular cell adhesion molecule-1, and intercellular adhesion molecule-1 in human endothelial cells. Blood 90: 1535–1544. 9269771

[5] Lafrenie RM, Wahl LM, Epstein JS, Hewlett IK, Yamada KM, et al (1996) HIV-1-Tat protein promotes chemotaxis and invasive behavior by monocytes. J Immunol 157: 974–977. 8757599

[6] Carlos TM, Harlan JM (1994) Leukocyte-endothelial adhesion molecules. Blood 84: 2068–2101. 7522621

[7] Meerschaert J, Furie MB (1995) The adhesion molecules used by monocytes for migration across endothelium include CD11a/CD18, CD11b/CD18, and VLA-4 on monocytes and ICAM-1, VCAM-1, and other ligands on endothelium. J Immunol 154: 4099–4112. 7535821

[8] Zietz C, Hotz B, Sturzl M, Rauch E, Penning R, et al (1996) Aortic endothelium in HIV-1 infection: chronic injury, activation, and increased leukocyte adherence. Am J Pathol 149: 1887–1898. 8952525 PMC1865334

[9] Dhawan S, Weeks BS, Soderland C, Schnaper HW, Toro LA, et al (1995) HIV-1 infection alters monocyte interactions with human microvascular endothelial cells. J Immunol 154: 422–432. 7527819

[10] Ambros V (2004) The functions of animal microRNAs. Nature 431: 350–355. 15372042 10.1038/nature02871

[11] Bartel DP (2004) MicroRNAs: genomics, biogenesis, mechanism, and function. Cell 116: 281–297. 14744438 10.1016/s0092-8674(04)00045-5

[12] Kloosterman WP, Plasterk RH (2006) The diverse functions of microRNAs in animal development and disease. Dev Cell 11: 441–450. 17011485 10.1016/j.devcel.2006.09.009

[13] Hu G, Gong AY, Liu J, Zhou R, Deng C, et al (2010) miR-221 suppresses ICAM-1 translation and regulates interferon-gamma-induced ICAM-1 expression in human cholangiocytes. Am J Physiol Gastrointest Liver Physiol 298: G542–550. 20110463 10.1152/ajpgi.00490.2009 PMC2853302

[14] Yao H, Yang Y, Kim KJ, Bethel-Brown C, Gong N, et al (2010) Molecular mechanisms involving sigma receptor-mediated induction of MCP-1: implication for increased monocyte transmigration. Blood 115: 4951–4962. 20354174 10.1182/blood-2010-01-266221 PMC2890169

[15] Reid W, Sadowska M, Denaro F, Rao S, Foulke J Jr, et al (2001) An HIV-1 transgenic rat that develops HIV-related pathology and immunologic dysfunction. Proc Natl Acad Sci U S A 98: 9271–9276. 11481487 10.1073/pnas.161290298 PMC55410

[16] Lin Z, Natesan V, Shi H, Hamik A, Kawanami D, et al (2010) A novel role of CCN3 in regulating endothelial inflammation. J Cell Commun Signal 4: 141–153. 21063504 10.1007/s12079-010-0095-x PMC2948121

[17] Lund AK, Lucero J, Lucas S, Madden MC, McDonald JD, et al (2009) Vehicular emissions induce vascular MMP-9 expression and activity associated with endothelin-1-mediated pathways. Arterioscler Thromb Vasc Biol 29: 511–517. 19150882 10.1161/ATVBAHA.108.176107 PMC4103743

[18] Hu G, Zhou R, Liu J, Gong AY, Eischeid AN, et al (2009) MicroRNA-98 and let-7 confer cholangiocyte expression of cytokine-inducible Src homology 2-containing protein in response to microbial challenge. J Immunol 183: 1617–1624. 19592657 10.4049/jimmunol.0804362 PMC2906382

[19] Chen XM, Splinter PL, O’Hara SP, LaRusso NF (2007) A cellular micro-RNA, let-7i, regulates Toll-like receptor 4 expression and contributes to cholangiocyte immune responses against Cryptosporidium parvum infection. J Biol Chem 282: 28929–28938. 17660297 10.1074/jbc.M702633200 PMC2194650

[20] Yao H, Peng F, Dhillon N, Callen S, Bokhari S, et al (2009) Involvement of TRPC channels in CCL2-mediated neuroprotection against tat toxicity. J Neurosci 29: 1657–1669. 19211873 10.1523/JNEUROSCI.2781-08.2009 PMC2768421

[21] Gendelman HE, Orenstein JM, Martin MA, Ferrua C, Mitra R, et al (1988) Efficient isolation and propagation of human immunodeficiency virus on recombinant colony-stimulating factor 1-treated monocytes. J Exp Med 167: 1428–1441. 3258626 10.1084/jem.167.4.1428 PMC2188914

[22] Yao H, Kim K, Duan M, Hayashi T, Guo M, et al (2011) Cocaine hijacks sigma1 receptor to initiate induction of activated leukocyte cell adhesion molecule: implication for increased monocyte adhesion and migration in the CNS. J Neurosci 31: 5942–5955. 21508219 10.1523/JNEUROSCI.5618-10.2011 PMC3410749

[23] Buckner CM, Calderon TM, Willams DW, Belbin TJ, Berman JW (2011) Characterization of monocyte maturation/differentiation that facilitates their transmigration across the blood-brain barrier and infection by HIV: implications for NeuroAIDS. Cell Immunol 267: 109–123. 21292246 10.1016/j.cellimm.2010.12.004 PMC4335637

[24] Westendorp MO, Frank R, Ochsenbauer C, Stricker K, Dhein J, et al (1995) Sensitization of T cells to CD95-mediated apoptosis by HIV-1 Tat and gp120. Nature 375: 497–500. 7539892 10.1038/375497a0

[25] Andras IE, Pu H, Deli MA, Nath A, Hennig B, et al (2003) HIV-1 Tat protein alters tight junction protein expression and distribution in cultured brain endothelial cells. J Neurosci Res 74: 255–265. 14515355 10.1002/jnr.10762

[26] Hayashi K, Pu H, Andras IE, Eum SY, Yamauchi A, et al (2006) HIV-TAT protein upregulates expression of multidrug resistance protein 1 in the blood-brain barrier. J Cereb Blood Flow Metab 26: 1052–1065. 16395283 10.1038/sj.jcbfm.9600254

[27] Prendergast MA, Rogers DT, Mulholland PJ, Littleton JM, Wilkins LH Jr, et al (2002) Neurotoxic effects of the human immunodeficiency virus type-1 transcription factor Tat require function of a polyamine sensitive-site on the N-methyl-D-aspartate receptor. Brain Res 954: 300–307. 12414113 10.1016/s0006-8993(02)03360-7

[28] Ho AW, Wong CK, Lam CW (2008) Tumor necrosis factor-alpha up-regulates the expression of CCL2 and adhesion molecules of human proximal tubular epithelial cells through MAPK signaling pathways. Immunobiology 213: 533–544. 18656701 10.1016/j.imbio.2008.01.003

[29] Huang WC, Chen JJ, Chen CC (2003) c-Src-dependent tyrosine phosphorylation of IKKbeta is involved in tumor necrosis factor-alpha-induced intercellular adhesion molecule-1 expression. J Biol Chem 278: 9944–9952. 12645577 10.1074/jbc.m208521200

[30] Lin FS, Lin CC, Chien CS, Luo SF, Yang CM (2005) Involvement of p42/p44 MAPK, JNK, and NF-kappaB in IL-1beta-induced ICAM-1 expression in human pulmonary epithelial cells. J Cell Physiol 202: 464–473. 15389584 10.1002/jcp.20142

[31] Ueda R, Kohanbash G, Sasaki K, Fujita M, Zhu X, et al (2009) Dicer-regulated microRNAs 222 and 339 promote resistance of cancer cells to cytotoxic T-lymphocytes by down-regulation of ICAM-1. Proc Natl Acad Sci U S A 106: 10746–10751. 19520829 10.1073/pnas.0811817106 PMC2705554

[32] Vittecoq D, Escaut L, Merad M, Teicher E, Monsuez JJ, et al (2003) Coronary heart disease in HIV-infected individuals. Adv Cardiol 40: 151–162. 14533552 10.1159/000073181

[33] Yunis NA, Stone VE (1998) Cardiac manifestations of HIV/AIDS: a review of disease spectrum and clinical management. J Acquir Immune Defic Syndr Hum Retrovirol 18: 145–154. 9637579 10.1097/00042560-199806010-00006

[34] Conaldi PG, Serra C, Dolei A, Basolo F, Falcone V, et al (1995) Productive HIV-1 infection of human vascular endothelial cells requires cell proliferation and is stimulated by combined treatment with interleukin-1 beta plus tumor necrosis factor-alpha. J Med Virol 47: 355–363. 8636703 10.1002/jmv.1890470411

[35] Grinspoon S, Carr A (2005) Cardiovascular risk and body-fat abnormalities in HIV-infected adults. N Engl J Med 352: 48–62. 15635112 10.1056/NEJMra041811

[36] Ren Z, Yao Q, Chen C (2002) HIV-1 envelope glycoprotein 120 increases intercellular adhesion molecule-1 expression by human endothelial cells. Lab Invest 82: 245–255. 11896203 10.1038/labinvest.3780418

[37] Fiala M, Popik W, Qiao JH, Lossinsky AS, Alce T, et al (2004) HIV-1 induces cardiomyopathyby cardiomyocyte invasion and gp120, Tat, and cytokine apoptotic signaling. Cardiovasc Toxicol 4: 97–107. 15371627 10.1385/ct:4:2:097

[38] Kline ER, Sutliff RL (2008) The roles of HIV-1 proteins and antiretroviral drug therapy in HIV-1-associated endothelial dysfunction. J Investig Med 56: 752–769. 10.1097/JIM.0b013e3181788d15 PMC2586126 18525451

[39] Krieglstein CF, Granger DN (2001) Adhesion molecules and their role in vascular disease. Am J Hypertens 14: 44S–54S. 11411765 10.1016/s0895-7061(01)02069-6

[40] Hope SA, Meredith IT (2003) Cellular adhesion molecules and cardiovascular disease. Part I. Their expression and role in atherogenesis. Intern Med J 33: 380–386. 12895171 10.1046/j.1444-0903.2003.00378.x

[41] Jang Y, Lincoff AM, Plow EF, Topol EJ (1994) Cell adhesion molecules in coronary artery disease. J Am Coll Cardiol 24: 1591–1601. 7963103 10.1016/0735-1097(94)90162-7

[42] Xia M, Ling W, Zhu H, Ma J, Wang Q, et al (2009) Anthocyanin attenuates CD40-mediated endothelial cell activation and apoptosis by inhibiting CD40-induced MAPK activation. Atherosclerosis 202: 41–47. 18495129 10.1016/j.atherosclerosis.2008.04.005

[43] Luo SF, Fang RY, Hsieh HL, Chi PL, Lin CC, et al (2010) Involvement of MAPKs and NF-kappaB in tumor necrosis factor alpha-induced vascular cell adhesion molecule 1 expression in human rheumatoid arthritis synovial fibroblasts. Arthritis Rheum 62: 105–116. 20039412 10.1002/art.25060

[44] Lund AK, Lucero J, Herbert L, Liu Y, Naik JS (2011) Human immunodeficiency virus transgenic rats exhibit pulmonary hypertension. Am J Physiol Lung Cell Mol Physiol 301: L315–326. 21685241 10.1152/ajplung.00045.2011 PMC3174744

[45] Friis-Moller N, Sabin CA, Weber R, d’Arminio Monforte A, El-Sadr WM, et al (2003) Combination antiretroviral therapy and the risk of myocardial infarction. N Engl J Med 349: 1993–2003. 14627784 10.1056/NEJMoa030218

[46] Friis-Moller N, Reiss P, Sabin CA, Weber R, Monforte A, et al (2007) Class of antiretroviral drugs and the risk of myocardial infarction. N Engl J Med 356: 1723–1735. 17460226 10.1056/NEJMoa062744

[47] Currier JS, Kendall MA, Henry WK, Alston-Smith B, Torriani FJ, et al (2007) Progression of carotid artery intima-media thickening in HIV-infected and uninfected adults. AIDS 21: 1137–1145. 17502724 10.1097/QAD.0b013e32811ebf79

[48] Holmberg SD, Moorman AC, Williamson JM, Tong TC, Ward DJ, et al (2002) Protease inhibitors and cardiovascular outcomes in patients with HIV-1. Lancet 360: 1747–1748. 12480430 10.1016/S0140-6736(02)11672-2

[49] Mondal D, Pradhan L, Ali M, Agrawal KC (2004) HAART drugs induce oxidative stress in human endothelial cells and increase endothelial recruitment of mononuclear cells: exacerbation by inflammatory cytokines and amelioration by antioxidants. Cardiovasc Toxicol 4: 287–302. 15470276 10.1385/ct:4:3:287

